# Associations of Intraoperative Hypotension and Vasopressor Administration With Postoperative Acute Kidney Injury in Children Undergoing Liver Transplantation: A Retrospective Cohort Study

**DOI:** 10.1111/pan.70090

**Published:** 2025-12-01

**Authors:** Theodora Wingert, Kelly Feldman, Tiffany Williams, Amelie Delaporte, Matthew Lum, Tristan Grogan, Christine Nguyen‐Buckley, Alexandre Joosten

**Affiliations:** ^1^ Department of Anesthesiology & Perioperative Medicine David Geffen School of Medicine at UCLA, University of California Los Angeles Los Angeles California USA; ^2^ Division of Critical Care Medicine, Department of Pediatrics University of California Los Angeles Los Angeles California USA; ^3^ Department of Anesthesiology, Perioperative and Pain Medicine Stanford University Stanford California USA; ^4^ Department of Medicine Statistics Core, David Geffen School of Medicine University of California Los Angeles Los Angeles California USA

**Keywords:** acute kidney injury, hypotension, liver transplantation, pediatric, postoperative complications, vasoconstrictor agents

## Abstract

**Background:**

Both intraoperative hypotension (IOH) and vasopressor administration are independently associated with postoperative acute kidney injury (AKI) in adults undergoing major noncardiac surgery. Whether these associations extend to children undergoing major noncardiac surgery, such as liver transplantation, remains unknown.

**Aims:**

This study aimed to evaluate whether IOH, defined as time spent with a mean arterial pressure (MAP) less than one standard deviation (SD) below age‐ and sex‐adjusted normal, and vasopressor administration in children are associated with postoperative AKI in liver transplantation (LT). We hypothesized that both IOH and vasopressor use would be independent predictors of postoperative AKI after pediatric LT.

**Methods:**

This single‐center retrospective cohort study analyzed all patients < 18 years undergoing LT, excluding those with preoperative end‐stage renal disease. The primary outcome was AKI, within 7 postoperative days defined according to KDIGO criteria. Multivariable logistic regression models were performed to determine whether IOH and vasopressor use, specifically maximum intraoperative epinephrine infusion, were independently associated with AKI. Exploratory K‐means clustering was applied to IOH and vasopressor exposure to identify hemodynamic phenotypes, which were evaluated for associations with AKI and other outcomes.

**Results:**

Of 144 pediatric LT cases, 22 were excluded for preexisting renal failure, leaving 122 for analyses. Postoperative AKI occurred in 39%. The mean cumulative duration MAP was < 1 standard deviation of age‐ and sex‐adjusted mean was 26.6 versus 26.1 min, respectively, among patients who developed AKI versus those who did not (mean difference 0.52 min: 95% CI −15.03, 16.07, *p* = 0.948). In the multivariate analysis neither hypotension (by the same definition) nor maximum epinephrine appeared to be associated with AKI: adjusted odds ratio 1.003 (95% CI: 0.992–1.014) and 1.003 (95% CI: 0.994–1.012). Exploratory cluster analysis revealed distinct intraoperative hemodynamic phenotypes based on IOH and vasopressor use, which were significantly associated with some perioperative outcomes, highlighting the need for larger studies with more robust control of patient factors.

**Conclusions:**

IOH and vasopressor exposure were not independently associated with AKI in children undergoing LT.

## 1 Introduction

While intraoperative hypotension (IOH) and vasopressor use are well‐established risk factors for acute kidney injury (AKI) and adverse postoperative outcomes in adult surgical populations, whether these associations apply to children remains unclear [1, 2, 3]. These associations have been observed to be particularly strong in adults undergoing high‐risk surgeries such as liver transplantation (LT) and open abdominal surgery [4, 5]. Existing pediatric studies of IOH have yielded inconsistent results, often limited by heterogeneous populations, variable definitions of hypotension, and low event rates [6, 7, 8]. Some studies in pediatric cardiac surgery have demonstrated associations with intraoperative mean arterial pressure (MAP) measurements and AKI, whereas the two large studies in noncardiac surgery have shown no association [6, 7, 8, 9, 10].

Pediatric LT presents unique hemodynamic challenges—including large fluid shifts, profound vasodilation, and post‐reperfusion instability—which may predispose patients to both hypotension and vasopressor exposure [11, 12, 13]. Additionally, AKI after pediatric LT occurs at rates much greater than other pediatric surgical cohorts, with rates ranging from 28% to 60% [14, 15, 16, 17]. Liver transplant patients have several unique physiologic risk factors for renal dysfunction that differentiate them from patients undergoing other major noncardiac surgery. These can range from preoperative dysfunction related to hepatorenal syndrome, ascites, or coagulopathy, to intraoperative factors such as reperfusion and hemodynamic instability, and postoperative factors such as nephrotoxic medications. Currently, the relationship between intraoperative factors and postoperative AKI in this setting remains poorly defined. Indeed, with the relatively high prevalence of IOH, vasopressor use, and AKI in pediatric LT, it may be an important subpopulation within pediatrics to study these relationships.

The primary aim of this study was to evaluate whether IOH (defined as time spent with a mean arterial pressure (MAP) less than one standard deviation (SD) below age‐ and sex‐adjusted normal) is independently associated with postoperative AKI in children undergoing LT. The secondary aim was to examine if intraoperative vasopressor administration was associated with postoperative AKI in children undergoing LT.

## 2 Methods

This single‐center retrospective cohort study was approved July 10, 2020 by the Ethics Committee of the University of California Los Angeles (IRB#20‐001083). We retrospectively evaluated all patients < 18 years who underwent LT at UCLA from 2013 to 2023. Patients with preoperative end‐stage renal disease, renal replacement therapy, and those undergoing simultaneous liver–kidney transplant were excluded from analyses with AKI as an outcome. The manuscript was prepared according to the STROBE guidelines.

### 2.1 Perioperative Characteristics

Patient demographics and perioperative variables were extracted from the electronic health record using our departmental Perioperative Data Warehouse [18]. Preoperative patient characteristics included age, sex, height, weight, American Society of Anesthesiologists (ASA) status, inpatient versus outpatient status, and laboratory values. Cause of liver failure was obtained from UNOS documentation. The presence of end‐stage renal disease (ESRD) was obtained from clinician‐entered data in the preoperative evaluation form and renal replacement therapy flowsheets. Intraoperative characteristics included type of anesthetic, duration of anesthesia, blood product transfusion, intraoperative medications administered, and intraoperative hemodynamic monitoring data. Note, for this study, we did not use the Pediatric End‐Stage Liver Disease (PELD) score because exception points, which are awarded outside the standard calculation and substantially influence listing severity, could not be reliably captured; instead, we incorporated the individual clinical components of PELD (including preoperative bilirubin, INR, creatinine, and preop dialysis) to ensure accurate representation of disease severity using objective data.

### 2.2 Hypotension

Intraoperative blood pressure values were recorded electronically automatically every minute with arterial blood pressure monitoring. Only arterial blood pressure monitoring was utilized to reduce data variation between noninvasive and arterial blood pressure measurements [19]. Artifacts were automatically removed from the raw blood pressure monitoring data to eliminate non‐physiologic measurements. Blood pressure was examined as cumulative duration (minutes) under systolic blood pressure (SBP) and mean arterial pressure (MAP) using age‐ and sex‐adjusted thresholds [6, 20]. Intraoperative blood pressure thresholds were defined according to the relationship to age‐ and sex‐adjusted reference values for children undergoing anesthesia during the preparation (also referred to commonly as the preincisional) phase [20]. Blood pressure values examined were those that occurred between the documented anesthesia start and anesthesia stop times. The total intraoperative time in minutes with blood pressure values under the aforementioned reference thresholds was calculated for each patient. For the purposes of this study, we defined intraoperative hypotension as the cumulative intraoperative duration with a mean arterial pressure less than one standard deviation below age‐adjusted normal.

### 2.3 Vasopressor Administration

Intraoperative vasopressor data were captured from the EHR. For the univariable analyses, we examined the maximum intraoperative vasopressor infusion rates of the four agents used by clinicians, including phenylephrine, norepinephrine, vasopressin, and epinephrine. For the two most common bolus agents, phenylephrine and norepinephrine, we examined total cumulative intraoperative doses administered per kilogram. For the purposes of the multivariable analyses, we utilized maximum intraoperative epinephrine infusion rate (μg/kg/min) as the primary measure of intraoperative vasopressor exposure, given its consistent use in this cohort.

Other studies have used various methods for defining IOH in children. Schacham et al. examined absolute lowest MAP and largest MAP reduction from individually derived baseline for 5 cumulative minutes [7]. At our institution, there is a high degree of missing and falsely elevated data with blood pressures obtained prior to induction; therefore this IOH definition was not used.

### 2.4 Outcomes

AKI was defined using the Kidney Disease: Improving Global Outcomes (KDIGO) criteria, as used by other studies [6, 7]. We utilized the most recent preoperative creatinine level as the baseline and the highest recorded postoperative creatinine within 7 days of surgery. The primary outcome was a dichotomous variable defined by the presence or absence of AKI. Patients were characterized into KDIGO stages of AKI according to whether they had a Cr increase of 1.5–1.9 times baseline or a ≥ 0.3 mg/dL absolute increase (KDIGO stage 1), 2.0–2.9 times baseline (KDIGO stage 2), or 3 times baseline or greater (KDIGO stage 3). Patients were also considered to have KDIGO stage 3 if Cr reached 4.0 mg/dL, the estimated glomerular filtration rate (GFR) dropped < 35 mL/min/1.73 m^2^, or new hemodialysis or new continuous renal replacement therapy (RRT) was initiated. For the purposes of this study, AKI was examined as a binary and categorical variable, with binary defined as KDIGO stage 1, 2, 3, or no AKI. Urine output criteria were not utilized due to a lack of consistent urine output data postoperatively.

Given that stage 1 KDIGO AKI is a mild and common phenomenon, often characterized only by an increase in creatinine with no clinically associated symptoms, we performed sensitivity analyses in which we specifically examined patients who developed KDIGO Stage 2 or 3 AKI. For these analyses, we redefined the primary outcome as only KDIGO Stage 2 or 3 AKI and compared these patients to (1) those without AKI, and (2) those without AKI or with Stage 1 AKI only. Given the limited sample size, only univariate comparisons were performed.

Length of stay (LOS) was measured as days between the surgery and discharge. Intensive care unit (ICU) LOS was defined as any ICU time after surgery before discharge from the ICU. Mortality was defined as any death occurring within the UCLA Health System, identified by discharge tables and death notes. Retransplant was defined according to history of prior liver transplant within institutional documentation. Surgeon years of training at the time of surgery was determined from the surgeon's last year of fellowship training in relationship to the year of surgery.

### 2.5 Anesthetic Management

At our institution, management of pediatric LT typically includes induction with intravenous agents and maintenance with volatile anesthetic and a fentanyl infusion. Patients undergo endotracheal intubation and placement of a radial arterial catheter, central venous catheter, and urinary catheter. Fluid resuscitation is at the discretion of the anesthesiologist with crystalloid, albumin, and blood products, and is guided by point‐of‐care laboratory testing. Vasoactive infusions are started when age‐appropriate blood pressure targets are not met despite adequate fluid and blood product administration, with epinephrine as the first‐line agent per institutional protocol. Vasopressors are also sometimes utilized to achieve a higher MAP goal post‐reperfusion. Surgical technique is at the discretion of the surgeon, with most cases involving total caval occlusion. Full data on venovenous bypass (VVB) were not available for the present study; however VVB is used rarely at our institution, and only in cases with a weight greater than 40 kg. At the conclusion of surgery, patients remain intubated, sedated, and are transported to the pediatric ICU for multidisciplinary care.

### 2.6 Statistical Methods

Patient characteristics and study variables were summarized overall and then by group (e.g., AKI stage or cluster group) using means with standard deviations (SD) for continuous variables and frequencies with percentages for categorical variables. Group comparisons were performed using *t*‐tests or ANOVA for continuous variables and chi‐square or Fisher's exact tests for categorical variables, as appropriate. Multivariable logistic regression was used to identify independent predictors of postoperative acute kidney injury (AKI), defined as any KDIGO stage versus none, with covariates selected based on clinical relevance. Given the small sample size, only a small number of variables were able to be included within the multivariable models. Results are presented as odds ratios (OR) with 95% confidence intervals (CI). Intraoperative hypotension for the purposes of this study was defined as the cumulative duration (minutes) of mean arterial pressure (MAP) less than 1 standard deviation (SD) below age‐ and sex‐adjusted thresholds, based on previously published pediatric norms [20]. Additionally, given that vasopressor selection preferences may change over time, we descriptively examined the rate of each vasopressor ever being used as an infusion intraoperatively for each year.

### 2.7 Exploratory Cluster Analysis

Exploratory analyses were undertaken to examine whether distinct hemodynamic phenotypes were observed with clustering based on cumulative hypotension duration and vasopressor exposure. K‐means clustering was applied to intraoperative hypotension and vasopressor exposure variables to determine whether distinct hemodynamic phenotypes could be identified [21, 22, 23]. Clustering inputs included cumulative hypotension durations and maximum infusion rates of phenylephrine, vasopressin, and epinephrine; the number of clusters (*k* = 3) was chosen based on interpretability and within‐cluster sum of squares. Outcomes across cluster groups were compared using ANOVA and Fisher's exact tests. To assess the stability of the clustering results, we performed k‐means clustering using 10 different random seeds and compared the resulting cluster assignments using the adjusted Rand index (ARI). An ARI of 1.0 indicates perfect agreement, while an ARI of 0 indicates agreement no better than random chance.

All analyses were conducted using R version 4.4.3 (R Foundation for Statistical Computing, Vienna, Austria). A two‐sided *p*‐value < 0.05 was considered statistically significant.

## 3 Results

Among the 144 pediatric LTs done over a 10‐year period (Table 1), 22 patients with preoperative end‐stage renal disease or renal replacement therapy were excluded from AKI analyses. This left 122 cases in the cohort for analyses. Patient characteristics according to postoperative AKI are seen in Table 1. The overall incidence of AKI (any KDIGO stage) was 39%, with stage 1 AKI in 20% of patients, stage 2 in 12%, and stage 3 in 4%. Inpatient mortality occurred in 4.2%. Retransplant rates were 3.5% at 30 days and 6.2% within the study period. The mean age was 6 years (median 3 years). Biliary atresia was the most common indication for transplant, accounting for almost one third of cases.

**TABLE 1:** Patient characteristics by AKI.

	Any AKI		
	No	Yes	*p*
	(*N* = 75)	(*N* = 47)	
Age	5.6 (5.6)	4.8 (5.1)	0.472
Weight (kg)	23.9 (21.2)	19.7 (20.3)	0.275
Male sex	39 (52%)	25 (53.2%)	0.898
ASA score			
2	1 (1.3%)	0	0.364
3	36 (48%)	18 (38.3%)	
4	38 (50.7%)	28 (59.6%)	
5	0	1 (2.1%)	
Emergent	11 (14.7%)	11 (23.4%)	0.222
Repeat LT	5 (6.7%)	4 (8.5%)	0.732
Anesthesia time (min)	482.1 (117.5)	505.8 (121.8)	0.288
Primary surgeon time (min)	291.6 (104.2)	329.6 (111.1)	0.059
Surgeon years of training	21.3 (10.1)	21.5 (7.9)	0.907
Preop GFR Schwartz	66.4 (26.0)	89.8 (45.1)	< 0.001
Preop creatinine (mg/dL)	0.4 (0.2)	0.4 (0.8)	0.736
Preop hemoglobin (g/dL)	10.2 (2.1)	9.9 (1.8)	0.418
Preop INR	1.5 (0.8)	1.6 (0.9)	0.639
Preop total bilirubin (mg/dL)	9.0 (11.6)	15.1 (16.7)	0.029
Preop ammonia (ug/dL)	134.0 (66.0)	193.1 (136.9)	0.135
UNOS cause of liver failure			0.067
Biliary atresia hypoplasia	24 (32%)	14 (29.8%)	
Acute hepatic necrosis	16 (21.3%)	2 (4.3%)	
Cirrhosis	10 (13.3%)	9 (19.1%)	
Metabolic disease	9 (12%)	10 (21.3%)	
Primary liver malignancy	11 (14.7%)	5 (10.6%)	
Other	5 (6.7%)	6 (12.8%)	
Intraop transfusion			
RBC (mL/kg)	23.6 (27.9)	35.2 (33.5)	0.040
FFP (mL/kg)	13.8 (25.6)	28.5 (40.7)	0.015
Platelet (mL/kg)	1.1 (4.1)	1.9 (5.6)	0.354
Cryoprecipitate (mL/kg)	1.1 (2.0)	1.6 (2.8)	0.277
Intraop vasopressor			
Phenylephrine total boluses (μg/kg)	133.9 (297.9)	191.1 (387.1)	0.361
Phenylephrine max rate (μg/min)	0.3 (1.7)	0.1 (0.9)	0.668
Vasopressin max rate (units/h/kg)	0.0 (0.0)	0.0 (0.0)	0.166
Epinephrine max (μg/kg/min)	0.0 (0.0)	0.0 (0.1)	0.042
Norepinephrine total boluses (μg/kg)	0.1 (0.3)	0.1 (0.3)	0.992
Norepinephrine max rate (μg/min)	2.2 (4.9)	2.0 (3.8)	0.767
Intraop arterial BP			
Duration SBP 1‐2SD	39.1 (47.6)	46.6 (40.4)	0.377
Duration SBP < 1SD	51.3 (64.2)	56.9 (50.7)	0.614
Duration SBP < 2SD	12.1 (25.3)	10.3 (14.5)	0.653
Duration MAP 1‐2SD	21.5 (36.5)	22.3 (35.4)	0.905
Duration MAP < 1SD	26.1 (42.7)	26.6 (41.4)	0.948
Duration MAP < 2SD	4.6 (9.3)	4.3 (7.9)	0.861
OUTCOMES			
Death (inpatient)	1 (1.3%)	2 (4.3%)	0.73
ICU LOS (h)	463.7 (429.8)	702.7 (861)	0.045
Hospital LOS (days)	48.3 (50.4)	58.7 (48.7)	0.269
Retransplant at 30 days	4 (5.3%)	1 (2.1%)	0.648
Retransplant (ever)	8 (10.7%)	1 (2.1%)	0.151
Postoperative RRT	0	7 (14.9%)	< 0.001

*Note:* Data are presented as mean (SD) for continuous variables and number (percent) for categorical variables.

Abbreviations: AKI, acute kidney injury; ASA, American Society of Anesthesiologists; FFP, fresh frozen plasma; ICU, intensive care unit; INR, international normalized ratio; intraop, intraoperative; LOS, length of stay; LT, liver transplantation; MAP, mean arterial pressure; Preop, preoperative; RBC, red blood cell; RRT, renal replacement therapy; SBP, systolic blood pressure.

The mean intraoperative minutes with MAP less than 1SD below age‐ and sex‐adjusted baseline was 26.1 min in those who did not develop AKI and 26.6 min in those who did develop AKI (mean difference 0.52 min: 95% CI −15.03, 16.07) (Table 1). Use of vasoactive drugs was also similar in the two groups. In the multivariate analysis neither hypotension (by the same definition) nor maximum epinephrine appeared to be associated with AKI: adjusted odds ratio 1.003 (95% CI: 0.992–1.014) and 1.003 (95% CI: 0.994–1.012) (Table 2).

**TABLE 2:** Multivariable adjusted analyses of AKI.

Variable	Any AKI		
	Odds ratio (OR)	95% CI (lower–upper)	*p*
Emergent	3.26	0.97–10.92	0.055
Primary surgeon time (min)	1.00	0.99–1.01	0.185
Weight (kg)	0.99	0.97–1.01	0.388
Epinephrine infusion max (ng/kg/min)	1.003	0.994–1.012	0.484
Preop GFR Schwartz	1.02	1.01–1.03	0.022
Preop total bilirubin (mg/dL)	1.05	1.01–1.09	0.011
Duration MAP < 1SD	1.003	0.992–1.014	0.637
Year of surgery	1.21	0.99–1.49	0.061
Surgeon years of training	1.06	0.99–1.12	0.084

Abbreviations: AKI, acute kidney injury; GFR, glomerular filtration rate; MAP, mean arterial pressure; SD, standard deviation.

As a sensitivity analysis, we redefined the primary outcome as only KDIGO Stage 2 or 3 AKI and compared these patients to those without AKI, and separately, to those without AKI or with Stage 1 only. Given the limited sample size, only univariate comparisons were performed and results were similar to the entire cohort analysis (Table S4). Additionally, given that vasopressor selection practice patterns and preferences may change over time, descriptive analyses showed that intraoperative norepinephrine and epinephrine infusions were more frequent during later years (Table S5).

Multivariable analyses also demonstrated preoperative GFR and total bilirubin to be associated with postoperative AKI (Table 2). Univariable and multivariable analyses of AKI KDIGO stage are seen in Tables S1 and S2. Multivariable analyses of AKI stage demonstrated emergent status, year of surgery, and surgeon years of training to be significantly associated with AKI stage in addition to preoperative GFR and total bilirubin (Table S2). Finally, while not the aims of the study, AKI KDIGO Stage was observed to be associated with ICU length of stay (LOS) in univariable analyses (Table 1).

**FIGURE 1:**
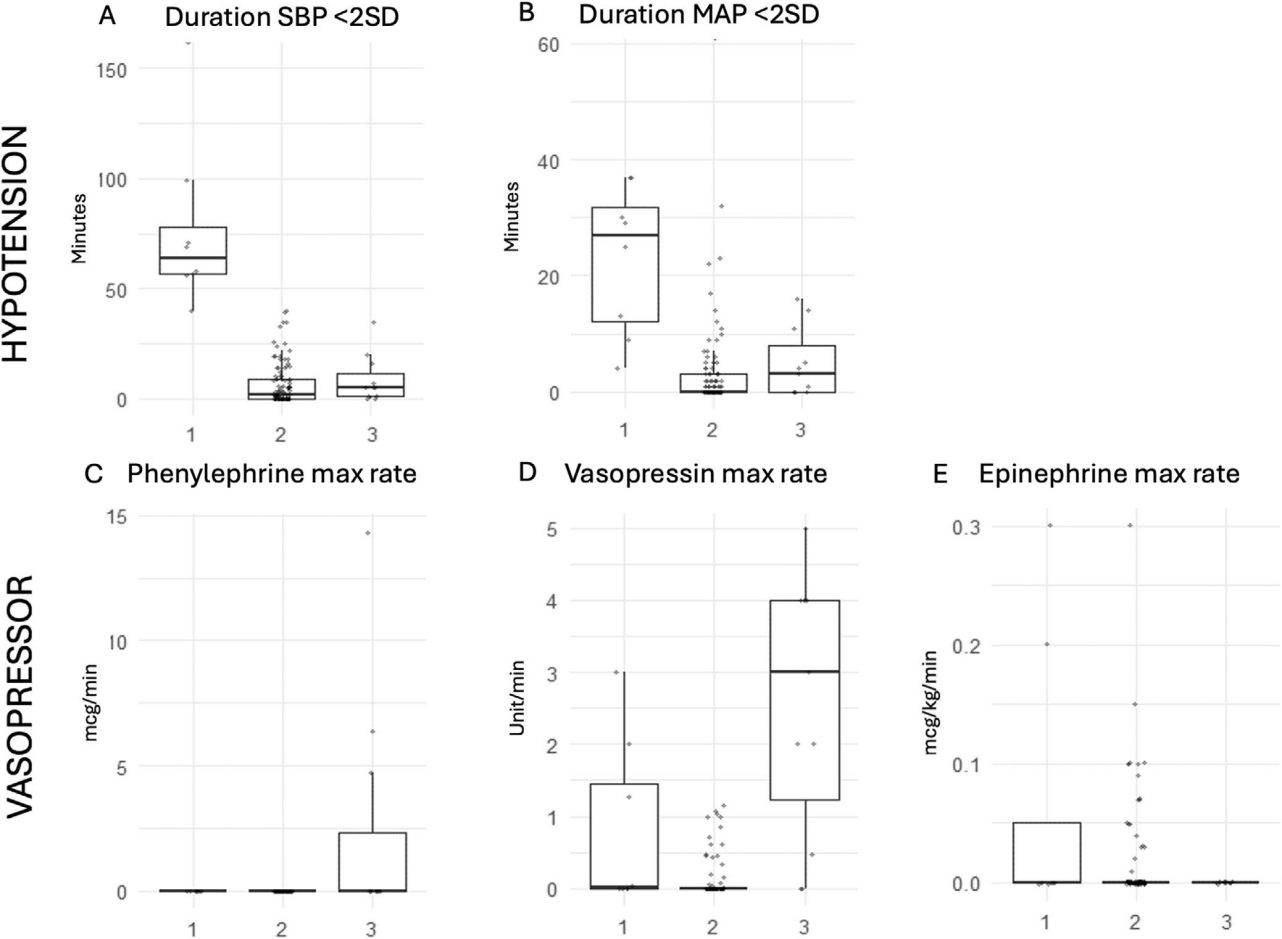
Box whisker plots of exploratory cluster groups. Given the small overall sample and individual cluster sizes, limited conclusions can be drawn from these analyses.

**FIGURE 2:**
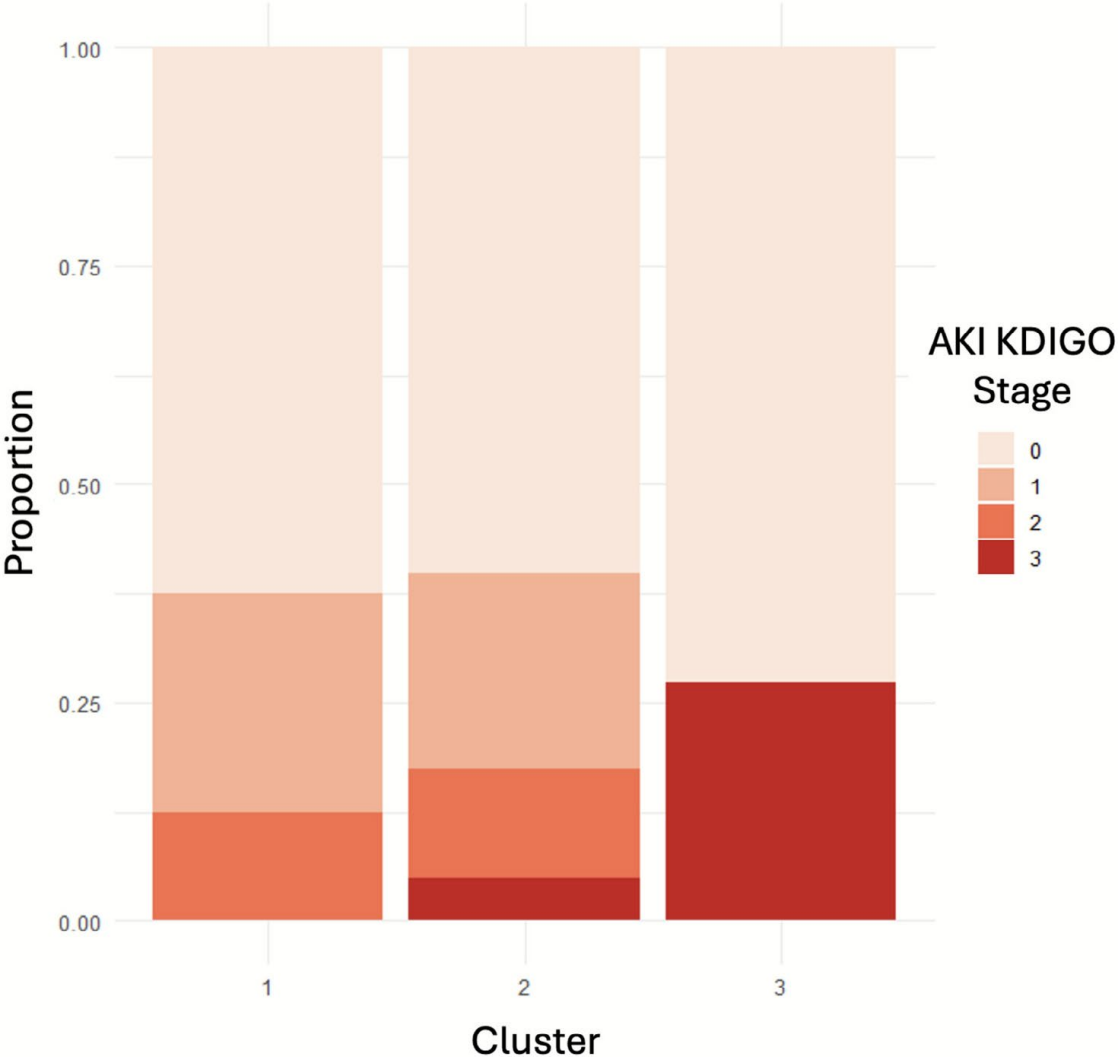
Distribution of AKI KDIGO stage according to exploratory cluster group. Given the small overall sample and individual cluster sizes, limited conclusions can be drawn from these analyses.

Exploratory k‐means clustering analysis demonstrated three unique clusters with clear differentiation according to intraoperative hypotension and vasopressor administration (Table S3, Figure 1). Cluster 2 had the lowest rates of both hypotension and vasopressor use; Cluster 1 had the longest periods of hypotension, paired with higher phenylephrine rates and low vasopressin and epinephrine use; and Cluster 3 had moderate rates of hypotension and high rates of vasopressin and epinephrine use (Figure 1). Notably, several patient characteristics were significantly different between the cluster groups (Table S3). The normotensive Cluster 2 had a median age of 2 years, whereas Cluster 1 and Cluster 3 had median ages of 6 and 18 years respectively. In univariable analyses, cluster group was not found to be associated with AKI—AKI occurred in 37.5%, 39.8%, and 27.3% of Cluster 1, Cluster 2, and Cluster 3 respectively (*p* = 0.804) (Figure 2). Of note, cluster phenotype was observed to be significantly associated with the need for postoperative hemodialysis or renal replacement therapy and hospital LOS (Table S3). Cluster 2 had an observed 3.9% rate of postoperative HD or RRT, while in Cluster 3 the observed rate was 27.3%, and none in Cluster 1 (*p* = 0.025), showing high rates of severe renal dysfunction in Cluster 3. Given the significant differences in each of the clusters with regard to age and other covariates, very limited conclusions can be made from these analyses.

To assess the stability of the clustering results, we performed k‐means clustering using 10 different random seeds and compared the resulting cluster assignments using the ARI. These analyses demonstrated that cluster stability was generally high, with a mean ARI of 0.81 (median: 1.00), suggesting that the overall structure of the clustering solution was reproducible across different initializations. However, some variability in patient assignments was observed in a subset of runs (minimum ARI: 0.44), which may reflect sensitivity to sample size and overlapping hemodynamic profiles.

## 4 Discussion

In this study of 122 children undergoing LT, IOH and vasopressor exposure were not independently associated with AKI in children undergoing LT. Postoperative AKI occurred in 39%, consistent with other studies. Exploratory cluster analyses revealed distinct hemodynamic phenotypes associated with adverse outcomes. These findings may be a starting point to better understand pediatric intraoperative hypotension and vasopressor administration and outcomes, both during LT and other high‐risk pediatric surgeries.

It is quite notable that while IOH and intraoperative vasopressors are associated with postoperative AKI in adults, these results have not been observed in this study and the small number of other pediatric studies examining this. The fact that AKI occurs much less frequently in pediatrics compared to adults makes it more difficult to study. Heterogeneity in age, weight, and surgical context may also dilute observable effects. However, there are several physiologically plausible reasons as well. There are differences in comorbidities, such as chronic hypertension and atherosclerosis, in addition to age‐associated changes in vascular stiffness. It is possible children may have different or more adaptable renal autoregulatory mechanisms that maintain renal perfusion despite fluctuations in blood pressure; however autoregulation of renal blood flow in pediatrics is poorly understood [24]. Finally, children typically have better baseline renal function and fewer preexisting renal insults, which may offer a greater reserve before renal insults occur.

While sample size is a serious limitation of this study, only about 500 pediatric LT are performed in the US per year [25]. As such, the sample size here is similar to other single‐center studies of pediatric LT [26]. Small sample sizes, missing or inaccurate data, and patient heterogeneity remain persistent challenges in pediatric research [27]. For example, consistent with other studies, urine output was not able to be factored in to AKI diagnosis, despite it being part of the diagnostic criteria [6, 7]. For this reason, in this study and others, we expect the reported AKI incidence is lower than the true incidence. Additionally, due to the small sample size, not all relevant variables were able to be included in the multivariable models. While there are several characteristics unique to LT, it remains an important high‐risk pediatric surgery to study given that postoperative AKI occurs fairly frequently in this cohort, as does IOH and vasopressor administration. As with adults, it is critical to study such high‐risk populations to elucidate subtle relationships or low incidence outcomes [4, 5, 10]. The liver transplant population however does have drawbacks in terms of variable clinical presentations and physiology specific to liver dysfunction and liver transplantation. Thus, the generalizability of extrapolating any findings in this population must be interpreted in light of this limitation.

Given that this study occurred over a 10‐year period, there were several notable temporal findings. We observed that both year of service and surgeon years of training at the time of surgery were associated with higher odds of AKI stage. These findings likely reflect unmeasured confounding, such as greater case complexity over time and by senior surgeons, and changes in practice patterns over time, such as reduced thresholds for the initiation of renal replacement therapy and intraoperative use of vasopressors. We also noted that vasopressor use varied over the time period, with norepinephrine and epinephrine both being used more frequently in later years.

In addition to all the above challenges specific to pediatric perioperative research, the study of pediatric IOH is also uniquely challenging. We chose to utilize age‐ and sex‐adjusted reference values due to the consistency of data with this methodology and lack of dependency on accurate preoperative blood pressure measurement. To date, there are only two large existing studies evaluating hypotension during pediatric noncardiac surgery [6, 7]. Numerous editorials bemoan the lack of consensus definition for intraoperative hypotension in pediatrics [28, 29]. In Schacham et al., hypotension was defined by the lowest MAP and the largest MAP reduction from baseline for at least 5 min. These traditional methods for defining hypotensive phenotype may not accurately characterize hypotension in pediatrics. In situations such as these, cluster analyses may provide insights and patterns not easily apparent with traditional statistics. Indeed, cluster analyses are being applied more often in clinical studies in recent years and have demonstrated the ability to elucidate phenotypes not recognizable by conventional analyses [21, 22, 23].

The association of vasopressors with AKI is another area where additional studies are needed. In adults, cumulative intraoperative vasopressors have been shown to be associated with AKI [3]. Understanding these relationships is critical for practicing physicians, who in the OR must decide whether to permit low blood pressure or utilize vasopressors to augment the blood pressure and cardiac output. In adults, there is evidence for higher vasopressor use being associated with higher rates of postoperative AKI [3].

In this single‐center retrospective analysis of intraoperative vasopressor and hypotension during pediatric LT, IOH and vasopressor exposure were not independently associated with postoperative AKI. Exploratory cluster analyses revealed distinct groups according to intraoperative hypotension and vasopressor administration that were significantly associated with length of stay and postoperative renal replacement therapy. While our exploratory cluster analysis identified distinct intraoperative hemodynamic phenotypes associated with length of stay and the need for postoperative renal replacement therapy, the clustering was not entirely robust across random seeds. This moderate instability, likely reflecting the small sample size and overlapping clinical characteristics, underscores the need for validation in larger cohorts. Cluster analyses may be helpful in future larger studies to better understand pediatric intraoperative hemodynamics. Further studies are needed in high‐risk groups such as pediatric liver transplant to better understand the patterns and the impact of intraoperative hypotension and vasopressor use.

## 5 Conclusion

In children undergoing liver transplantation, IOH and vasopressor exposure were not independently associated with postoperative AKI. Exploratory cluster analyses revealed distinct intraoperative hemodynamic phenotypes linked to adverse outcomes; however limited conclusions can be made given the small sample size. These results offer an exploratory lens to which we hope may stimulate future efforts to better characterize the relationship between intraoperative hemodynamics and postoperative outcomes in pediatric LT and other high‐risk surgeries.

## Supporting information


**Data S1:** Supporting Information

## Data Availability

The data that support the findings of this study are available on request from the corresponding author. The data are not publicly available due to privacy or ethical restrictions.
