# Supplementary material for: Associations of Intraoperative Hypotension and Vasopressor Administration With Postoperative Acute Kidney Injury in Children Undergoing Liver Transplantation: A Retrospective Cohort Study
Source: Paediatr Anaesth. 2025 Dec 1;36(3):240–8. doi: 10.1111/pan.70090 (PMC12887151; doi:10.1111/pan.70090)
Supplement: Supplementary file 1 — Data S1: Supporting Information [file PAN-36-240-s001.pdf]

Supplemental Table 1 Patient Characteristics by AKI KDIGO Stage

		AKI Class				
	**Overall (N=144)**	**0 (N=75)**	**1 (N=25)**	**2 (N=14)**	**3 (N=8)**	**P-value**
Age	5.9 (5.8)	5.6 (5.6)	5.2 (5.1)	4.2 (4.9)	5.0 (6.1)	0.846
Weight (kg)	24.4 (21.9)	23.9 (21.2)	18.4 (12.8)	17.3 (11.8)	27.9 (42.2)	0.443
Male sex	77 (53.5%)	39 (52.0%)	13 (52.0%)	8 (57.1%)	4 (50.0%)	0.989
ASA Score						
2	1 (0.7%)	1 (1.3%)	0	0	0	0.298
3	58 (40.3%)	36 (48.0%)	12 (48.0%)	5 (35.7%)	1 (12.5%)	
4	81 (56.2%)	38 (50.7%)	12 (48.0%)	9 (64.3%)	7 (87.5%)	
5	4 (2.8%)	0	1 (4.0%)	0	0	
Emergent	32 (22.2%)	11 (14.7%)	2 (8.0%)	5 (35.7%)	4 (50.0%)	0.014
Repeat LT	9 (6.2%)	5 (6.7%)	1 (4.0%)	2 (14.3%)	1 (12.5%)	0.442
Anesthesia time (min)	494.1 (118.6)	482.1 (117.5)	540.0 (128.9)	452.2 (91.2)	492.6 (122.6)	0.105
Primary surgeon time (min)	305.8 (104.2)	291.6 (104.2)	358.3 (127.3)	284.1 (84.5)	319.4 (71.1)	0.046
Preop GFR Schwartz	73.3 (38.4)	66.4 (26.0)	91.8 (39.1)	105.3 (44.0)	56.3 (52.6)	<0.001
Preop Creatinine (mg/dL)	0.5 (0.7)	0.4 (0.2)	0.3 (0.4)	0.2 (0.1)	1.2 (1.6)	<0.001
Preop Hemoglobin (g/dL)	9.8 (2.0)	10.2 (2.1)	9.9 (1.8)	10.0 (2.2)	9.5 (1.6)	0.78
Preop INR	1.6 (0.9)	1.5 (0.8)	1.6 (1.1)	1.4 (0.6)	1.9 (0.8)	0.669
Preop Total Bilirubin (mg/dL)	12.9 (14.2)	9.0 (11.6)	14.1 (12.9)	11.9 (12.9)	22.6 (27.4)	0.048
Preop Ammonia (ug/dL)	190.6 (115.4)	134.0 (66.0)	210.7 (150.6)	114.5 (58.3)	350.0 ( )	0.071
UNOS Cause of Liver Failure						0.118
Biliary atresia hypoplasia	42 (29.2%)	24 (32.0%)	6 (24.0%)	6 (42.9%)	2 (25.0%)	
Acute Hepatic Necrosis	23 (16.0%)	16 (21.3%)	1 (4.0%)	0	1 (12.5%)	
Cirrhosis	23 (16.0%)	10 (13.3%)	6 (24.0%)	1 (7.1%)	2 (25.0%)	
Metabolic disease	22 (15.3%)	9 (12.0%)	5 (20.0%)	3 (21.4%)	2 (25.0%)	
Primary liver malignancy	18 (12.5%)	11 (14.7%)	2 (8.0%)	3 (21.4%)	0	
Other	16 (11%)	5 (6.7%)	5 (20.0%)	1 (7.1%)	1 (12.5%)	
INTRAOP TRANSFUSION						
RBC (ml/kg)	34.1 (42.4)	23.6 (27.9)	32.7 (30.8)	37.8 (38.2)	38.5 (36.7)	0.207
FFP (ml/kg)	25.8 (44.1)	13.8 (25.6)	25.4 (31.6)	34.0 (56.2)	28.9 (38.6)	0.091
Platelet (ml/kg)	1.8 (4.9)	1.1 (4.1)	0.8 (2.4)	2.9 (7.2)	3.7 (9.0)	0.263
Cryoprecipitate (ml/kg)	1.5 (2.5)	1.1 (2.0)	1.2 (3.0)	2.2 (2.5)	1.7 (2.3)	0.397
INTRAOP VASOPRESSOR						

Phenylephrine total boluses (mcg/kg)	7.7 (16.9)	6.6 (12.9)	5.5 (7.8)	3.5 (6.3)	26.5 (42.8)	0.004
Phenylephrine max rate (mcg/min)	0.4 (2.8)	0.3 (1.7)	0.0 (0.0)	0.0 (0.0)	0.8 (2.2)	0.553
Vasopressin max rate (units/hr/kg)	0.0 (0.0)	0.0 (0.0)	0.0 (0.0)	0.0 (0.0)	0.0 (0.1)	0.544
Epinephrine infusion max (mcg/kg/min)	0.0 (0.0)	0.0 (0.0)	0.0 (0.1)	0.0 (0.0)	0.0 (0.0)	0.003
Norepinephrine total boluses (mcg/kg)	0.1 (0.3)	0.1 (0.3)	0.2 (0.4)	0.0 (0.1)	0.0 (0.0)	0.448
Norepinephrine max rate (mcg/min)	3.0 (7.0)	2.2 (4.9)	1.8 (3.0)	0.9 (1.8)	4.4 (6.9)	0.357
INTRAOP ARTERIAL BP						
Duration SBP 1-2SD	38.2 (43.9)	39.1 (47.6)	49.7 (34.0)	30.4 (33.4)	65.0 (61.2)	0.255
Duration SBP <1SD	50.4 (61.0)	51.3 (64.2)	59.3 (41.6)	38.7 (45.2)	81.1 (76.1)	0.402
Duration SBP <2SD	12.2 (27.9)	12.1 (25.3)	9.6 (13.8)	8.4 (15.3)	16.1 (15.6)	0.824
Duration MAP 1-2SD	19.9 (34.3)	21.5 (36.5)	16.8 (18.1)	24.5 (51.0)	35.8 (44.4)	0.625
Duration MAP <1SD	25.3 (45.0)	26.1 (42.7)	19.9 (21.0)	30.6 (61.9)	40.8 (47.2)	0.647
Duration MAP <2SD	5.4 (17.5)	4.6 (9.3)	3.1 (6.3)	6.1 (11.3)	5.0 (5.3)	0.767
OUTCOMES						
Death (inpatient)	6 (4.2%)	1 (1.3%)	1 (4.0%)	0	1 (12.5%)	0.393
ICU LOS (hours)	609.4 (767.0)	463.7 (429.8)	668.7 (984.4)	536.8 (346.0)	1099.5 (1043.2)	0.042
Hospital LOS (days)	56.3 (57.8)	48.3 (50.4)	53.4 (51.7)	55.9 (38.8)	83.1 (55.0)	0.358
Retransplant at 30days	5 (3.5%)	4 (5.3%)	0	0	1 (12.5%)	0.354
Retransplant (ever)	9 (6.2%)	8 (10.7%)	0	0	1 (12.5%)	0.181
Postoperative RRT	22 (15.3%)	0	0	0	7 (87.5%)	<0.001

Data are presented as mean (SD) for continuous variables and number (percent) for categorical variables. Abbreviations: Acute kidney injury (AKI), American Society of Anesthesiologists (ASA), Liver transplant (LT), Preoperative (Preop), International normalized ratio (INR), Intraoperative (intraop), systolic blood pressure (SBP), mean arterial pressure (MAP), Length of Stay (LOS), Intensive Care Unit (ICU), Renal Replacement Therapy (RRT).

Supplemental Table 2. Multivariable adjusted analyses of AKI KDIGO Stage.

	AKI KDIGO Stage		
Variable	Odds Ratio (OR)	95% CI (Lower–Upper)	p-value
Emergent	4.60	1.68 – 12.63	0.003
Primary surgeon time (min)	1.002	0.998 – 1.006	0.338
Weight (kg)	0.99	0.98 – 1.02	0.882
Epinephrine infusion max (mcg/kg/min)	1.00	0.99 – 1.01	0.946
Preop GFR Schwartz	1.01	1.00 – 1.02	0.032
Preop Total Bilirubin (mg/dL)	1.05	1.02 – 1.08	0.003
Duration MAP <1SD	1.003	0.99 – 1.01	0.484
Year of Surgery	1.18	1.18 – 1.18	<0.001
Surgeon Years of Training	1.05	1.00 – 1.11	0.044

Abbreviations: Acute kidney injury (AKI), glomerular filtration rate (GFR), standard deviation (SD), confidence interval (CI), Preoperative (Preop), mean arterial pressure (MAP).

Supplemental Table 3. Intraoperative Hypotension and Vasoactive Cluster Group Characteristics

	Cluster			
	1	2	3	P-value
	(N=8)	(N=103)	(N=11)	
Age				
Mean	6.2 (4.7)	4.4 (4.8)	12.7 (5.8)	<0.001
Median	6.0 (2.6, 9.8)	2.0 (1.0, 6.0)	16.0 (8.0, 17.0)	
Weight (kg)	22.9 (17.6)	19.0 (16.5)	52.3 (34.6)	<0.001
Male sex	4 (50.0%)	51 (49.5%)	9 (81.8%)	0.129
ASA				0.787
2	0	1 (1.0%)	0	
3	4 (50.0%)	44 (42.7%)	6 (54.5%)	
4	4 (50.0%)	57 (55.3%)	5 (45.5%)	
5	0	1 (1.0%)	0	
Emergent	0	20 (19.4%)	2 (18.2%)	0.543
Repeat LT	1 (12.5%)	7 (6.8%)	1 (9.1%)	0.424
Patient Class				0.025
Emergency	0	2 (1.9%)	2 (18.2%)	
Inpatient	7 (87.5%)	100 (97.1%)	9 (81.8%)	
Same Day Admit	1 (12.5%)	0	0	
Outpatient	0	1 (1.0%)	0	
Primary surgeon time (min)	318.5 (83.3)	301.5 (110.8)	342.0 (97.1)	0.474
Preop GFR Schwartz	71.4 (43.2)	78.1 (36.1)	53.9 (28.7)	0.103
Preop Creatinine	0.4 (0.3)	0.3 (0.2)	1.2 (1.3)	<0.001
OUTCOMES				
Any AKI	3 (37.5%)	41 (39.8%)	3 (27.3%)	0.804
AKI KDIGO Stage				
None	5 (62.5%)	62 (60.2%)	8 (72.7%)	0.085
1	2 (25.0%)	23 (22.3%)	0	
2	1 (12.5%)	13 (12.6%)	0	
3	0	5 (4.9%)	3 (27.3%)	
Death (inpatient)	0	3 (2.9%)	0	1.000
Length of Stay (days)				
Mean	102.6 (125.9)	48.9 (36.9)	46.5 (51.7)	0.011
Median	39.5 (32.0, 110.2)	36.0 (24.0, 62.0)	16.0 (13.0, 56.5)	
ICU LOS (hours)	1065.6 (1595.4)	512.9 (439.2)	591.0 (982.4)	0.061
Postop Hemodialysis or RRT	0	4 (3.9%)	3 (27.3%)	0.025
INTRAOP				
RBC (ml/kg)	62.3 (56.4)	25.5 (26.0)	26.5 (34.1)	0.004
FFP (ml/kg)	59.0 (63.6)	16.1 (27.7)	21.8 (32.7)	0.001
Platelet (ml/kg)	4.5 (5.1)	1.3 (4.9)	0.3 (1.1)	0.137
Cryoprecipitate (ml/kg)	3.3 (3.8)	1.1 (2.1)	1.4 (2.5)	0.033
Phenylephrine total Bolus (mcg/kg)	19.5 (23.3)	5.3 (13.1)	17.5 (24.9)	0.003
Phenylephrine max rate (mcg/min)	0.0 (0.0)	0.0 (0.0)	2.3 (4.6)	<0.001

Vasopressin bolus (units/kg)	0.1 (0.2)	0.0 (0.1)	0.1 (0.1)	<0.001
Vasopressin max rate (units/hr/kg)	0.0 (0.1)	0.0 (0.0)	0.1 (0.0)	<0.001
Epinephrine infusion max (mcg/kg/min)	0.1 (0.1)	0.0 (0.0)	0.0 (0.0)	0.015
Epinephrine total bolus (mcg/kg)	0.4 (0.6)	0.5 (1.4)	0.4 (1.0)	0.976
Norepinephrine total bolus (mcg/kg)	0.0 (0.1)	0.1 (0.3)	0.2 (0.5)	0.402
Norepinephrine max rate (mcg/min)	2.7 (3.7)	1.2 (2.5)	10.1 (9.7)	<0.001
Lowest intraop Hemoglobin	6.8 (1.9)	7.4 (1.3)	7.9 (1.3)	0.192
Duration SBP <0SD	259.9 (84.7)	148.9 (107.5)	178.8 (91.7)	0.015
Duration SBP 0-1SD	95.9 (39.0)	105.0 (71.2)	116.6 (52.5)	0.796
Duration SBP 1-2SD	87.5 (39.9)	37.2 (43.2)	53.5 (47.4)	0.006
Duration SBP <2SD	76.5 (38.5)	6.7 (9.5)	8.6 (10.9)	<0.001
Duration SBP <1SD	164.0 (69.4)	43.9 (49.8)	62.2 (54.4)	<0.001
Duration MAP <0SD	239.8 (109.1)	105.8 (97.5)	142.0 (87.2)	<0.001
Duration MAP 0-1SD	140.9 (60.8)	85.9 (74.2)	116.2 (68.5)	0.067
Duration MAP 1-2SD	78.0 (68.1)	17.6 (30.0)	20.4 (22.2)	<0.001
Duration MAP <2SD	20.9 (14.2)	3.1 (7.0)	5.5 (6.6)	<0.001
Duration MAP <1SD	98.9 (72.8)	20.7 (35.1)	25.8 (24.9)	<0.001

Data are presented as mean (SD) for continuous variables and number (percent) for categorical variables, unless specifically noted. Abbreviations: Acute kidney injury (AKI), American Society of Anesthesiologists (ASA), Liver transplantation (LT), Preoperative (Preop), International normalized ratio (INR), Intraoperative (intraop), systolic blood pressure (SBP), mean arterial pressure (MAP), Length of Stay (LOS), Intensive Care Unit (ICU).

Supplemental Table 4 Sensitivity Analyses

	AKI KDIGO Stage 2/3 vs No AKI			AKI KDIGO Stage 2/3 vs Stage 1 AKI/No AKI		
	No AKI (N=75)	AKI 2/3 (N=22)	P-value	No AKI or Stage 1 AKI (N=100)	AKI 2/3 (N=22)	P-value
Age	5.6 (5.6)	4.5 (5.2)	0.419	5.5 (5.4)	4.5 (5.2)	0.443
Weight (kg)	23.9 (21.2)	21.1 (26.6)	0.609	22.5 (19.6)	21.1 (26.6)	0.774
Male sex	39 (52.0%)	12 (54.5%)	0.833	52 (52.0%)	12 (54.5%)	0.829
ASA Score						
2	1 (1.3%)	0	0.176	1 (1.0%)	0	0.283
3	36 (48.0%)	6 (27.3%)		48 (48.0%)	6 (27.3%)	
4	38 (50.7%)	16 (72.7%)		50 (50.0%)	16 (72.7%)	
5	0	0		1 (1.0%)	0	
Emergent	11 (14.7%)	9 (40.9%)	0.014	13 (13.0%)	9 (40.9%)	0.005
Repeat LT	5 (6.7%)	3 (13.6%)	0.376	6 (6.0%)	3 (13.6%)	0.205
Anesthesia time (min)	482.1 (117.5)	466.9 (102.7)	0.585	496.6 (122.4)	466.9 (102.7)	0.293
Primary surgeon time (min)	291.6 (104.2)	297.0 (80.1)	0.825	308.3 (113.5)	297.0 (80.1)	0.658
Preop GFR Schwartz	66.4 (26.0)	87.5 (52.0)	0.011	72.8 (31.7)	87.5 (52.0)	0.088
Preop Creatinine (mg/dL)	0.4 (0.2)	0.6 (1.0)	0.19	0.4 (0.3)	0.6 (1.0)	0.104
Preop Hemoglobin (g/dL)	10.2 (2.1)	9.8 (2.0)	0.485	10.1 (2.0)	9.8 (2.0)	0.548
Preop INR	1.5 (0.8)	1.6 (0.7)	0.752	1.5 (0.9)	1.6 (0.7)	0.86
Preop Total Bilirubin (mg/dL)	9.0 (11.6)	16.2 (20.1)	0.049	10.2 (12.1)	16.2 (20.1)	0.086
Preop Ammonia (ug/dL)	134.0 (66.0)	161.6 (116.8)	0.506	161.6 (108.1)	161.6 (116.8)	1
UNOS Cause of Liver Failure						
Biliary atresia hypoplasia	24 (32.0%)	8 (38.1%)	0.615	30 (30.0%)	8 (38.1%)	0.83
Acute Hepatic Necrosis	16 (21.3%)	1 (4.8%)		17 (17.0%)	1 (4.8%)	
Cirrhosis	10 (13.3%)	3 (14.3%)		16 (16.0%)	3 (14.3%)	
Metabolic disease	9 (12.0%)	5 (23.8%)		14 (14.0%)	5 (23.8%)	
Primary liver malignancy	11 (14.7%)	3 (14.3%)		13 (13.0%)	3 (14.3%)	
Other	5 (6.7%)	2 (9.1%)		10 (10%)	2 (9.1%)	
INTRAOP TRANSFUSION						
RBC (ml/kg)	23.6 (27.9)	38.1 (36.7)	0.049	25.8 (28.8)	38.1 (36.7)	0.089
FFP (ml/kg)	13.8 (25.6)	32.1 (49.6)	0.022	16.7 (27.5)	32.1 (49.6)	0.046
Platelet (ml/kg)	1.1 (4.1)	3.2 (7.7)	0.095	1.0 (3.7)	3.2 (7.7)	0.051
Cryoprecipitate (ml/kg)	1.1 (2.0)	2.0 (2.4)	0.073	1.1 (2.3)	2.0 (2.4)	0.099
INTRAOP VASOPRESSOR						
Phenylephrine total boluses (mcg/kg)	6.6 (12.9)	11.8 (27.6)	0.212	6.3 (11.8)	11.8 (27.6)	0.139

Phenylephrine max rate (mcg/min)	0.3 (1.7)	0.3 (1.4)	0.93	0.2 (1.5)	0.3 (1.4)	0.777
Vasopressin max rate (units/hr/kg)	0.0 (0.0)	0.0 (0.0)	0.225	0.0 (0.0)	0.0 (0.0)	0.374
Epinephrine infusion max (mcg/kg/min)	0.0 (0.0)	0.0 (0.0)	0.525	0.0 (0.1)	0.0 (0.0)	0.212
Norepinephrine total boluses (mcg/kg)	0.1 (0.3)	0.0 (0.0)	0.266	0.1 (0.3)	0.0 (0.0)	0.188
Norepinephrine max rate (mcg/min)	2.2 (4.9)	2.2 (4.6)	0.971	2.1 (4.5)	2.2 (4.6)	0.952
INTRAOP ARTERIAL BP						
Duration SBP 1-2SD	39.1 (47.6)	43.0 (47.2)	0.741	41.8 (44.7)	43.0 (47.2)	0.912
Duration SBP <1SD	51.3 (64.2)	54.1 (60.3)	0.853	53.3 (59.3)	54.1 (60.3)	0.951
Duration SBP <2SD	12.1 (25.3)	11.2 (15.5)	0.866	11.5 (22.9)	11.2 (15.5)	0.951
Duration MAP 1-2SD	21.5 (36.5)	28.6 (48.0)	0.459	20.3 (32.9)	28.6 (48.0)	0.33
Duration MAP <1SD	26.1 (42.7)	34.3 (56.1)	0.464	24.6 (38.4)	34.3 (56.1)	0.326
Duration MAP <2SD	4.6 (9.3)	5.7 (9.4)	0.627	4.2 (8.6)	5.7 (9.4)	0.475
OUTCOMES						
Death (inpatient)	1 (1.4%)	1 (4.5%)	0.405	2 (2.0%)	1 (4.5%)	0.453
ICU LOS (hours)	463.7 (429.8)	741.4 (716.7)	0.027	515.5 (618.8)	741.4 (716.7)	0.135
Hospital LOS (days)	48.3 (50.4)	65.0 (45.4)	0.175	49.6 (50.5)	65.0 (45.4)	0.2
Retransplant at 30days	4 (5.3%)	1 (4.5%)	1	4 (4.0%)	1 (4.5%)	1
Retransplant (ever)	8 (10.7%)	1 (4.5%)	0.679	8 (8.0%)	1 (4.5%)	1

Data are presented as mean (SD) for continuous variables and number (percent) for categorical variables.
Abbreviations: Acute kidney injury (AKI), American Society of Anesthesiologists (ASA), Liver transplant (LT), Preoperative (Preop), International normalized ratio (INR), Intraoperative (intraop), systolic blood pressure (SBP), mean arterial pressure (MAP), Length of Stay (LOS), Intensive Care Unit (ICU).

Supplemental Table 5 Intraoperative Vasopressor Infusion use over time

Year of service	N cases	Norepinephrine	Epinephrine	Vasopressin	Phenylephrine	Dopamine	Milrinone
2013	17	17.6%	5.9%	17.6%	0%	0%	0%
2014	13	0%	0%	15.4%	15.4%	0%	0%
2015	13	15.4%	15.4%	7.7%	0%	0%	0%
2016	7	14.3%	28.6%	28.6%	0%	0%	0%
2017	12	58.3%	0%	16.7%	0%	0%	0%
2018	16	75.0%	31.2%	37.5%	0%	0%	0%
2019	7	42.9%	85.7%	28.6%	0%	0%	0%
2020	14	64.3%	28.6%	64.3%	14.3%	0%	0%
2021	10	60.0%	50.0%	60.0%	0%	0%	0%
2022	13	76.9%	30.8%	46.2%	7.7%	0%	0%
